# Pelvic floor and abdominal muscle cocontraction in women with and without pelvic floor dysfunction: a systematic review and meta-analysis

**DOI:** 10.6061/clinics/2019/e1319

**Published:** 2019-11-19

**Authors:** Giovana Vesentini, Regina El Dib, Leonardo Augusto Rachele Righesso, Fernanda Piculo, Gabriela Marini, Guilherme Augusto Rago Ferraz, Iracema de Mattos Paranhos Calderon, Angélica Mércia Pascon Barbosa, Marilza Vieira Cunha Rudge

**Affiliations:** I Departamento de Ginecologia e Obstetricia, Faculdade de Medicina de Botucatu, Universidade Estadual Paulista (UNESP), Botucatu, SP, BR; II Departamento de Biociencias e Diagnostico Bucal, Instituto de Ciencia e Tecnologia, Universidade Estadual Paulista (UNESP), Sao Jose dos Campos, SP, BR; III McMaster Institute of Urology, McMaster University, Hamilton, Ontario, Canada; IV Department of Oral and Maxillofacial Surgery, Mainz University Medical Center, Mainz, Germany; V Departamento de Ciencias da Saude, Universidade Sagrado Coracao, Bauru, SP, BR; VI Departamento de Fisioterapia e Terapia Ocupacional, Universidade Estadual Paulista (UNESP), Marilia, SP, BR

**Keywords:** Pelvic Floor Disorders, Abdominopelvic Muscles, Pelvic Floor Function, Synergism

## Abstract

There is an ongoing discussion regarding abdominal muscle (AbM) and pelvic floor muscle (PFM) synergism. Therefore, this study aimed to investigate the cocontraction between AbMs and PFMs in women with or without pelvic floor dysfunction (PFD). The following databases were searched up to December 21, 2018: MEDLINE, EMBASE, LILACS, PEDro and CENTRAL. We included any study that assessed the cocontraction between PFMs and AbMs in women with and without PFD. Two reviewers independently screened eligible articles and extracted data. The outcomes were extracted and analyzed as continuous variables with random effect models. Twenty studies were included. A meta-analysis did not show differences in women with and without PFD. However, a sensitivity analysis suggested cocontraction of the transversus abdominis (TrA) during PFM contraction in healthy women (standardized mean difference (SMD) –1.02 [95% confidence interval (CI) −1.90 to −0.14], P=0.02; I^2^= not applicable; very low quality of evidence). Women with PFD during contraction of PFMs showed cocontraction of the obliquus internus (OI) (SMD 1.10 [95% CI 0.27 to 1.94], P=0.01; I^2^= not applicable; very low quality of evidence), and obliquus externus (OE) (SMD 2.08 [95% CI 1.10 to 3.06], P<0.0001; I^2^ = not applicable; very low quality of evidence). Increased cocontraction of the TrA may be associated with maximal contraction of PFMs in women without PFD. On the other hand, there is likely an increased cocontraction with the OI and OE in women with PFD.

## INTRODUCTION

Pelvic floor dysfunction (PFD) refers to a group of disturbances in the pelvic floor muscles (PFM) or connective tissues usually associated with pelvic organ prolapse, urinary and/or anal incontinence, sexual dysfunction, and pelvic pain (1). Treatment-related costs are estimated to correspond to an annual expenditure of 12 billion dollars and are projected to increase every year (2), with a considerable prevalence according to the population and definition used (3). The estimated prevalence is reported to be 25% to 46% in high-income (4), low-income and middle-income countries (5). PFD is a common disease that affects women at all ages, exerting a severe impact on their lives and consuming considerable healthcare resources (4).

Researchers have reported strategies, such as the use of a model of abdominal muscle (AbM) training to stimulate tonic PFM activity (6). This scientific evidence is based on the idea of synergistic cocontraction of the PFMs and AbMs, which occurs during normal activities (7,8). Although there is an established literature highlighting that PFM and AbM interaction is usually present in asymptomatic women (9), clinical practice guidelines for conservative management of PFD (10,11) have demonstrated that the AbMs remain a neglected aspect of care. The addition of AbM training might improve clinical outcomes for patients with PFD (12) and restore normal PFM function. The lack of establishment of coactivation between PFMs and AbMs in women with PFD might reflect the lack of robust evidence that exercise regimens other than PFM training would potentially add benefits to conservative management of PFD (13).

The understanding of cocontraction among AbMs and PFMs could be valuable for alternative strategies of PFM exercises to promote continence. In this systematic review, we investigate the coactivity of AbMs – transversus abdominis (TrA), rectus abdominis (RA), obliquus internus (OI), and obliquus externus (OE) – and PFMs in women with or without PFD. We hypothesized that women with PFD would show decreased coactivity of the AbMs or PFMs during maximal voluntary contraction (MVC) of the PFMs or AbMs, respectively, compared to women with no history of PFD.

## MATERIALS AND METHODS

This review adhered to the Preferred Reporting Items for Systematic Reviews and Meta-analyses - PRISMA (14) and Meta-analysis of Observational Studies in Epidemiology - MOOSE (15) guidelines and was registered on PROSPERO (CRD42017055462).

### Eligibility criteria

- Study design: any observational study (cohort, cross-sectional, comparative cross-sectional) or any baseline subset of data provided by randomized controlled trials, to avoid interaction effects due to any applied interventions. Studies that aimed to assess the reliability of scoring systems for the investigation of cocontraction of the muscles under investigation in this review, as well as studies that provided information on our predefined outcomes, were also included;
- Participants: women with or without PFD, with urinary incontinence (UI), pelvic organ prolapse (POP), and pelvic pain;
- Interventions: any voluntary contraction of PFMs that recorded the cocontraction of AbMs (TrA, RA, OI, and OE) and vice versa;
- Outcomes:
-
  - The cocontraction of AbMs (TrA, RA, OI, and OE) and PFMs was measured by surface electromyography (EMG), ultrasonography (US), a digital palpation scale, or a perineometer;

We also considered any indirect assessment of the muscle contraction.

We excluded full-text peer-review studies that evaluated AbMs and PFMs in resting activity.

### Data source and searches

Using the Medical Subject Headings (MeSH), based on the combination of terms “female urinary incontinence,” “continent,” “pelvic floor,” “abdominopelvic musculature,” and “abdominal muscle,” we ran the search strategy in MEDLINE (1980 to December 21, 2018), EMBASE (1980 to December 21, 2018), PEDro (1999 to December 21, 2018), LILACS (1982 to December 21, 2018), and CENTRAL (1999 to December 21, 2018). No language restriction was applied. This strategy was similar for the other databases and was executed until December 21, 2018 (Appendix).

### Selection of studies

Two reviewers (GV and LARR) independently screened all titles and abstracts identified by the literature search, obtained full-text articles of all potentially relevant records, and evaluated them. Disagreements were resolved through discussion or by consulting a third person (RED).

### Data extraction

Data from included studies were summarized in a standardized data extraction with participant demographics, inclusion and exclusion criteria, cocontraction measurement methods, muscles studied and outcomes. Two reviewers (GV and LARR) extracted the sample size, means and standard deviations (SD). When SD data were unavailable, we estimated the SD using the standard error according to the recommendations of the Cochrane Handbook (16).

If data regarding methods or results were incomplete, we attempted to contact the authors for further information. Moreover, when we found figures without data, we used the WebPlotDigitizer^®^ (v. 3.8) for Windows to extract an estimation of the data from the figures.

### Risk of bias assessment

The risk of bias with a modified version of the Ottawa-Newcastle instrument was independently assessed by the reviewers (17). This tool includes confidence in the assessment of exposure and outcome and an adjusted analysis for differences between groups in prognostic characteristics and missing data (17). When information regarding risk of bias or other aspects of methods or results was unavailable, we attempted to contact the study authors for additional information.

### Certainty of evidence

The Grading of Recommendations Assessment, Development and Evaluation (GRADE) system was used to rate the certainty of the evidence for each outcome measure as high, moderate, low, or very low (18). Detailed GRADE guidance was performed according to the following criteria: imprecision (19), inconsistency (20), and indirectness (21). The results are summarized in a table of evidence profile.

### Data synthesis and statistical analysis

We analyzed the outcomes as continuous variables with random effect models on the results from the muscles investigated (TrA, RA, OI, and OE). Since the assessment of cocontraction in the included studies was measured in different ways (e.g., US and EMG), the individual scales were aligned to point in the same direction, and we calculated the standardized mean difference (SMD) along with the respective confidence interval (CI) of 95%, using the extracted means and SDs (16). Positive SMD values indicated higher cocontraction of the evaluated muscle in the PFD group compared to the asymptomatic group, and a negative SMD indicated higher cocontraction of the evaluated muscle in the asymptomatic group compared to the PFD group.

We also conducted sensitivity analyses to test the robustness of these results. When data were obtained from RCTs and the results were provided separately by intervention and control groups, we calculated the baseline mean and SD based on the mean and SD from the studies. Furthermore, when studies provided both the left and right sides of the AbMs, we also calculated the mean and SD based on the mean and SD provided for both sides.

We calculated the heterogeneity across studies using the I^2^ statistic and the *p*-value for the Chi-square test using Review Manager software (RevMan version 5.3; Nordic Cochrane Center, Cochrane).

## RESULTS

### Search results

**Figure 1:**
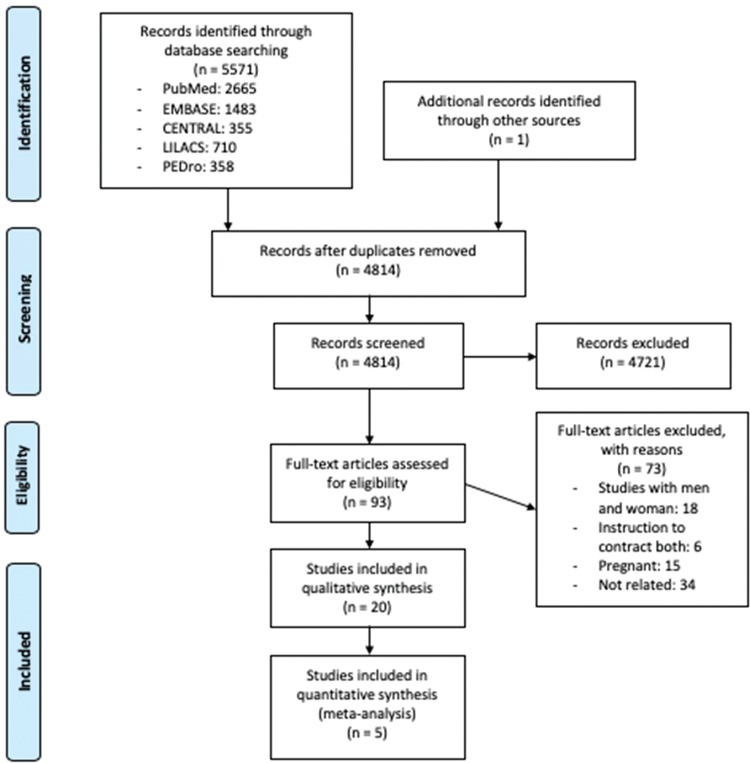
Flowchart of the studies included in this review.

Figure 1 presents the PRISMA flow diagram for identifying eligible studies based on title and abstract screening. After the assessment of 93 full texts, we included 20 studies included in the systematic review with a subset of data provided by one RCT (22), one prospective (23) and 18 cross-sectional studies (8,9,24-39) with a total of 468 participants. The interobserver agreement for screening was substantial (kappa 0.82).

### Study characteristics

The sample size of the studies ranged from three (26) to 44 (31) participants. Typical participants were aged from 19 (25) to 66 (34) years old (Table 1). From a total of 20 included studies, four (8,9,32,39) recorded the activity of all AbMs (TrA, RA, OI, and OE) during PFM contraction, and 19 studies provided instructions to contract the PFMs and recorded the AbM coactivity (8,9,22-35,37-39). Fifteen studies (8,9,22-24,26,28,31-37,39) reported the MVC of the PFMs. Three studies (23,27,34) considered the standing position for the assessment of the coactivity, and another eleven studies considered the supine position (8,9,22,25,29-31,35-38). Four studies (28,32,33,39) considered different positions – standing, sitting and supine, and one did not report the position for the assessment of coactivity (26). Fifteen studies (8,9,23,25,26,28,30-35,37-39) measured the contraction by EMG, four studies (22,24,27,36) measured the contraction by US, and one study (29) measured the contraction by visual inspection and digital palpation scale (Table 2).

### Risk of bias assessment

**Figure 2:**
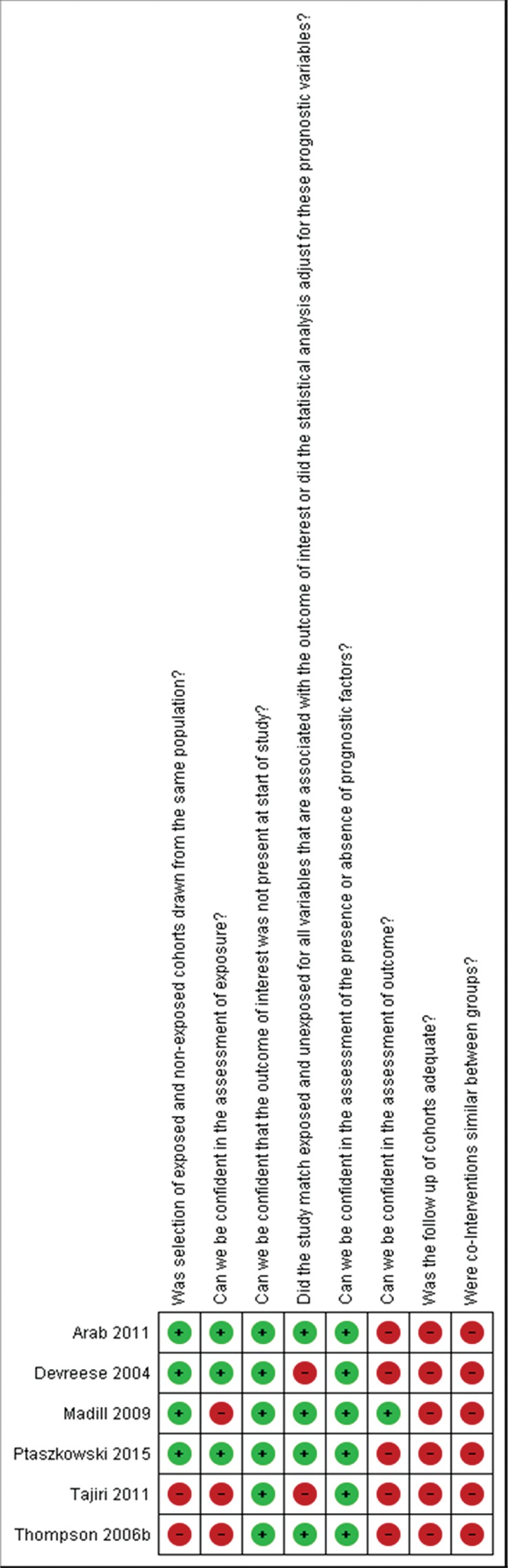
Risk of bias assessment. We considered “probably high risk of bias” as “definitely high risk of bias” (red color) and “probably low risk of bias” as “definitely low risk of bias” (green color).

Figure 2 describes the risk of bias summary of the studies that compared two groups. Six observational studies compared women with and without PFD. The main problems with the studies were follow-up (24,29,31,34,36,38), information regarding cointerventions (24,29,31,34,36,38), assessment of outcome (24,29,34,36,38) and exposure (31,36,38). Table 3 details the description for each study.

### Outcomes

#### Meta-analysis of TrA muscle cocontraction when the PFMs contract

**Figure 3:**
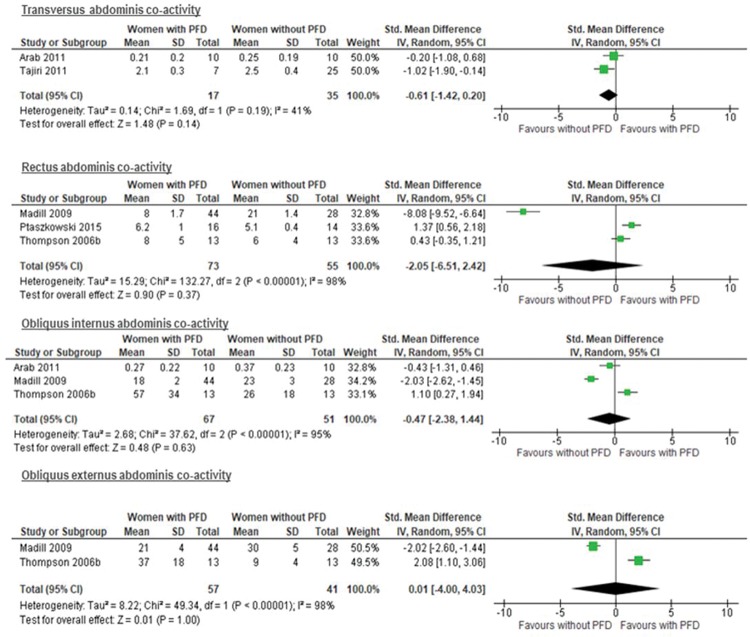
Forest plot showing the co-activity of the transversus abdominis, rectus abdominis, obliquus internus and obliquus externus muscles during maximal pelvic floor muscle contraction. CI = Confidence interval; PFD = Pelvic floor dysfunction.

**Figure 4:**
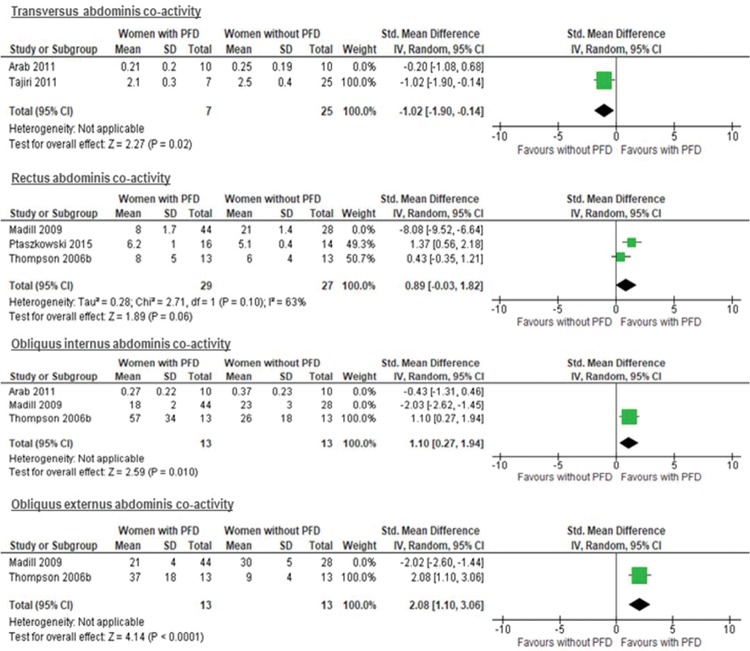
Sensitivity analysis of co-activity of transversus abdominis (without the Arab et al. 2011 study), rectus abdominis (without the Madill et al. (31)), obliquus internus (without the Madill et al. (31)) and obliquus externus (without the Madill et al. (31)) muscles when the pelvic floor muscles contract. CI = Confidence interval; PFD = Pelvic floor dysfunction.

The results from two studies (24,36) with a total of 52 participants assessing cocontraction by US failed to show a difference in the cocontraction of the TrA in women with and without PFD (SMD −0.61 [95% CI −1.41 to 0.20], *p*=0.14; I^2^= 41%) (Figure 3). However, a plausible sensitivity analysis, excluding the study of Arab et al. (24), yielded results that were inconsistent with the primary analysis, showing higher coactivity of the TrA during MVC of the PFMs in women without PFD (SMD −1.02 [95% CI −1.90 to −0.14], *p*=0.02; I^2^= not applicable) (Figure 4).

Certainty evidence was rated down to low because of serious limitations on the high risk of bias, indirectness due to the evaluation of only one PFD (UI) (Figure 3) and different ages, as well as imprecision (Table 4).

#### Meta-analysis of RA muscle cocontraction when the PFMs contract

The results from three studies (31,34,38) with a total of 128 participants were unable to demonstrate a difference in the cocontraction of the RA between women with a normal pelvic floor and women with PFD (UI) (SMD −2.05 [95% CI −6.51 to 2.42], P=0.37; I^2^= 98%) (Figure 3). Furthermore, the sensitivity analysis, excluding the Madill et al. study (31), showed results that were inconsistent with the primary analysis, with higher cocontraction of the RA during MVC of the PFMs in women with PFD, however, with no statistical significance (SMD 0.89 [95% CI -0.03 to 1.82], P=0.06; I2= 63%) (Figure 4).

Certainty of evidence was rated down to very low because of serious limitations on the high risk of bias, inconsistency due to high heterogeneity (Figure 3), indirectness due to evaluation of only one PFD (UI), different assessments of UI and different ages, and imprecision (Table 4).

#### Meta-analysis of OI abdominis muscle cocontraction when the PFMs contract

The results from three studies (24,31,38) with a total of 118 participants showed no difference between women with a normal pelvic floor and women with PFD (UI) (SMD −0.47 [95% CI −2.38 to 1.44], I^2^= 95%; P=0.63) (Figure 3). However, a plausible sensitivity analysis, excluding the studies of Madill et al. (31) and Arab et al. (24), presented results that were inconsistent with the primary analysis, showing a higher mean of cocontraction in women with PFD (UI) than in women with a normal pelvic floor (SMD 1.10 [95% CI 0.27 to 1.94], P=0.01; I2= not applicable) (Figure 4).

Certainty of evidence was rated down to very low because of serious limitations on inconsistency due to high risk of bias, high heterogeneity (Figure 3), indirectness due to the evaluation of only one PFD (UI), different assessments of UI and different ages, and imprecision (Table 4).

#### Meta-analysis of OE abdominis muscle cocontraction when the PFMs contract

The results from two studies (31,38) with a total of 98 participants failed to show a difference between women with a normal pelvic floor and women with PFD (SMD 0.01 [95% CI −4.00 to 4.03], P=1.00; I^2^= 98%) (Figure 3). However, a plausible sensitivity analysis, excluding the study of Madill et al. (31), demonstrated results that were inconsistent with the primary analysis, showing a higher mean of cocontraction in women with PFD (UI) than in women with a normal pelvic floor (SMD 2.08 [95% CI 1.10 to 3.06], P<0.0001; I2= not applicable) (Figure 4).

Certainty of evidence was rated down to very low because of serious limitations on inconsistency due to high heterogeneity (Figure 3), indirectness due to high risk of bias, evaluation of only one PFD (UI), different assessments of UI and different ages, and imprecision (Table 4).

## DISCUSSION

### Main findings

This systematic review that investigated the cocontraction of AbMs and PFMs in women with or without PFD identified 20 studies. Therefore, it might provide evidence of synergism between PFMs and the TrA, RA, OI and OE, i.e., the cocontraction of PFMs and AbMs occurs during both voluntary contraction of the pelvic floor and abdominal muscle contractions. The studies showed a cocontraction of AbMs during the contraction of PFMs in women with no history of symptoms of PFD, with PFD, or both. Meta-analysis of data from five cross-sectional studies assessed the synergism of the TrA, RA, OI, and OE during MVC of PFMs. As the primary meta-analysis failed to show any difference between women with and without PFD, we performed a sensitivity analysis to minimize the heterogeneity of data. Our sensitivity analysis showed a different cocontraction pattern according to the four AbMs considered. The cocontraction between the TrA and PFMs in asymptomatic women showed a higher activation than that in symptomatic women. However, compared to women without PFD, women with PFD, such as UI, demonstrated an increased cocontraction of AbMs (RA, OI, and OE), suggesting an altered mechanism.

One study (24) was excluded for a sensitivity analysis on the cocontraction of the TrA and OI because it did not report the position of women during the measurement. Additionally, as prior to the testing, the participants were trained until the correct performance of PFM contraction, we believe that such training before the measurement may have affected the data provided. Furthermore, another study (31) was not included in a sensitivity analysis of RA, OI, and OE. Although this study had the highest sample size, women with PFD were classified as having mild or severe UI, according to the severity of urine leakage. Moreover, the EMG data provided were smoothed by computing the root mean square. In this sensitivity analysis of RA, the I^2^ value, previously at 100%, was reduced to 0% when this study (31) was removed. Moreover, the results from the sensitivity analysis in OI and OE reached statistical significance favoring the PFD group.

### Strengths and limitations

The strengths of our study include our unique analysis of the influence of each of the four muscles from the abdominal wall during maximal and submaximal contraction of PFMs. Additionally, we have provided evidence of a different synergism between AbMs and PFMs in women with and without PFD.

The primary limitation of our review is the low evidence because of study limitations. We identified a small number of studies with a small number of participants, resulting in high CIs; therefore, these findings should be carefully interpreted. EMG results should be cautiously interpreted because most studies used surface electrodes, which may contaminate data and distort their interpretation because of the surrounding muscles (40). Additionally, the data processing of EMG studies widely differs, mostly in the position of the electrodes, the position of evaluation, and the type of data normalization.

Another limitation of this review was the insufficient number of included studies; we were not able to perform the complete statistical analysis. Furthermore, publication bias was not assessed because there were <10 eligible studies for each outcome in the meta-analysis (16).

### Relation to prior work

Although previous systematic reviews have shown evidence of cocontraction between PFMs and AbMs (41,42), investigators had not previously conducted a comparison between women with a normal pelvic floor and those with PFD involving all four muscles of the abdominal wall (TrA, RA, OI, and OE). Furthermore, to our knowledge, there is no published meta-analysis of the cocontraction between PFMs and the four AbMs.

The first systematic review related to this theme focused only on the combined training of the TrA and PFMs to treat UI and included five studies (41). Another previous systematic review focused only on healthy women and included ten studies (42). In contrast, our search found 20 studies, and only five could be included in the meta-analyses. Our much larger analyses, including 468 women, more precisely elucidated the biomechanics of the communication between the abdominopelvic muscles in both the normal pelvic floor and PFD. Furthermore, we have also been able to detect the influence of each of the four muscles of the abdominal wall in PFM contraction.

### Implications

PFD is very common among women worldwide and has become an increasing socioeconomic problem with prejudicial public health consequences, including symptoms that could lead to a significant decrease in quality of life and disability (43). While the prevalence of PFD is high, many factors involved in PFD are often poorly recognized or understood. Knowing the pathways related to PFD in detail is a main goal facilitating the identification of tools to prevent or correct these disorders (44). Our findings suggest a mechanism of PFD that is related to changes in the biomechanics caused by the increased AbM activation strength or by recruitment timing activation associated with different coactivity mechanisms according to the AbMs and PFMs.

In our view, there is a plausible biomechanical explanation to support higher coactivation levels of AbMs during MVC of PFMs. The coactivation between the TrA and PFMs showed a higher activation in asymptomatic women than in symptomatic women. However, the pattern of activation of the other AbMs differs with respect to time and strength in symptomatic women. During muscle contraction in PFD, there is a rapid and stronger coactivity of the RA, OI, and OE. The stronger coactivity of these AbMs could cause an increase in intra-abdominal pressure that, added to the insufficient PFM contraction, would increase the PFD.

Pereira et al. (45) proposed a theory explaining the synergism between the TrA and PFM. The abdominopelvic cavity has a static function of containment of the viscera and interacts with the PFMs. The fibers from the TrA are prolonged by the transverse perineal muscle because these muscles belong to the same muscle chain. This is an important conclusion for rehabilitation therapy, since numerous studies focus only on TrA strengthening to induce greater contractile strength of PFMs (22,27,35,36). Knowledge of the synergism among PFMs and AbMs may be useful for assessing PFMs and teaching women how to perform PFM exercises.

Our results show a synergism between AbMs and PFMs in women with and without PFD in different positions of evaluation. However, the studies included in this review had no standardized methods for selecting the participants, sample size, EMG, and US measurement, which limits the reliability of the findings. Very low-quality evidence suggests an association between the cocontraction of the AbMs when PFMs contract either in women with a normal pelvic floor or in women with PFD and should be interpreted with caution. Further research is needed to provide a better understanding of the cocontraction between the PFMs and AbMs.

## AUTHOR CONTRIBUTIONS

Vesentini G, El Dib R and Rudge MVC were involved in the conception and design of the review. Vesentini G and El Dib R developed the search strategy. Vesentini G and Righesso LAR performed the study selection and data collection. Vesentini G, El Dib R, Righesso LAR and Rudge MVC were involved in the data analysis. Vesentini G, El Dib R, Rudge MVC and Barbosa AMP were involved in the interpretation and discussion of results. Vesentini G drafted the manuscript, and El Dib R, Piculo F, Marini G, Ferraz GAR, Calderon IMP, Barbosa AMP and Rudge MVC contributed to the drafting of the review. All authors approved the final version of the manuscript for publication.

## APPENDIX

**Table:** Search strategy.

(Women OR woman OR female OR Women’s Groups OR Women’s Group OR Women Groups OR Women Group OR healthy women OR healthy woman OR incontinent OR incontinent women OR incontinent woman OR urinary incontinence in women OR Female Urinary Incontinence OR continent OR continent women OR continent woman OR urgency urinary incontinence OR Urinary Stress Incontinence OR stress urinary incontinence OR stress urinary OR UUI OR SUI OR MUI OR Urinary Urge Incontinence OR Urinary Reflex Incontinence OR Urge Incontinence OR mixed urinary incontinence OR Urinary Bladder Disease OR Urinary Bladder Diseases OR Urinary Reflex Incontinence) AND ((Pelvic Floor OR Pelvic Diaphragm OR Pelvic Diaphragms OR Pelvic Floor Disorders OR Pelvic Floor Disorder OR Pelvic Floor Disease OR Pelvic Floor Diseases OR pelvic floor dysfunction OR pelvic floor dysfunctions OR Pelvic Floor muscle OR Pelvic Floor muscles OR Urinary Incontinence OR abdomino-pelvic musculature OR perineal musculature OR Perineum OR perineums OR perineal function OR pelvic floor contraction OR pelvic floor muscle contractions OR co-contraction OR muscle synergism OR muscle co-contraction OR co-activity OR co-activity muscle) AND (Abdominal Muscles OR Abdominal Muscle OR Abdomen OR Abdomens OR abdomino-pelvic musculature OR transversus abdominis OR Rectus Abdominis OR Rectus Muscle of Abdomen OR Abdomen Rectus Muscle OR Abdomen Rectus Muscles OR external obliques OR external oblique OR internal obliques OR internal oblique OR abdominal muscle contractions OR synergistic co-contraction of abdominal muscles OR synergism co-contraction of abdominal muscles OR co-contraction OR muscle synergism OR muscle co-contraction OR co-activity OR co-activity muscle))

## Figures and Tables

**Table 1:** Study characteristics related to the study design, location, number of participants, mean age, and inclusion and exclusion criteria.

Author	Study design	Location	No. participants	Mean age	Inclusion criteria	Exclusion criteria
**With Pelvic Floor Dysfunction**						
Bø et al. (27)	Cross-sectional	Europe	13	46.5	Consecutive women, at their first consultation in an ongoing randomized clinical trial on PFMT to reduce POP	Inability to understand the Norwegian language and contract the PFMs; nulliparous or less than 12 m pp; previous pelvic surgery; chronic lung disease, or stage 0 and 4 POP measured by the POP quantified
Tajiri et al. (22)	Randomized control trial	Asia	15	52	Women who had experienced one or more SUI events in the last 1 m	NR
**Without Pelvic Floor Dysfunction**						
Bø et al. (26)	Cross-sectional	Europe	3	31.6	Physical therapists; aged 30-33 y; extensive experience in correct PFM contractions	NR
Bø et al. (25)	Cross-sectional	Europe	6	19.5	Women aged 19-21 y; nulliparous; no history of UI, neurological disease, or urinary tract infection; exercising regularly more than 3×/week	NR
Sapsford et al. (6)	Cross-sectional	Australia	7	49.3	Parous women with a history of vaginal deliveries	History of PFD or LBP; abdominal or pelvic surgery; neurological or respiratory condition; regularly performing sit-ups or AbM training
Neumann et al. (33)	Cross-sectional	Australia	4	34	Nulliparous women aged 25-42 y, who were tested on two occasions 1 week apart	Skinfold thickness of >2.5 cm; history of LBP; known or suspected pregnancy; UI; urinary tract or vaginal infection; surgery involving incision of the left abdominal wall
Madill et al. (9)	Cross-sectional	Canada	15	36.3	Continent women aged 21- 60 y; not pregnant; had not given birth in the previous 12 months; in good general health	History of DM, neurological conditions, or autoimmune CT disorders; used any medications to treat or known to exacerbate UI; previous history of SUI
Thompson et al. (37)^a^	Cross-sectional	Australia	13	37	Women aged 20-55 y and premenopausal or on HRT and consistent PFM exercise technique	History of urinary tract or vaginal infection; known or suspected pregnancy; surgery involving incision of the abdominal wall; obesity; history of LBP or sporting activities; neurological disorders; inability to understand English
Madill et al. (32)	Cross-sectional	Canada	15	36.3	Women aged 21-60 y; no history of SUI; not pregnant or had not given birth in the previous 12 m and in good general health	History of DM; neurological conditions; autoimmune CT disorders; used any medications to treat or known to exacerbate UI; history of SUI
Junginger et al. (30)	Cross-sectional	NR	9	42	Volunteers without PFM disorders; aged 32-59 y; with height of 157-174 cm and weight of 57-72 kg	History of LBP; hip or abdominal surgery or history of PFD and of laparotomy
Strupp et al. (35)	Cross-sectional	Central and South America	34	28.1	Willingness to participate in the study and ability to contract the PFM and perform the AHT correctly	Unable to contract AbM and PFM correctly; pregnancy; neurological disease; autoimmune CT disorder or PFD
Chmielewska et al. (28)	Cross-sectional	Europe	19	23.6	Continent women aged 19-28 y	SUI; pregnancy; childbirth(s); pelvic surgery; DM; hypertension; neurological abnormalities; urinary tract infection; elevated temperature; practicing a professional sport; spinal pain; obesity
Silva et al. (23)	Prospective	Central and South America	25	24.76	Women aged 18-35 y; no history of UI	Virgin women; abdominal-pelvic surgeries; metabolic disorders; presence of myopathies and collagen diseases, neurological disorders, cognitive disturbance and physical limitations; previous PFM training; inability to contract PFM
Ithamar et al. (39)	Cross-sectional	Central and South America	30	25.77	women aged 18-35 y; BMI between 18.50-24.99 kg/m^2^; abdominal skinfold ≤3 cm; with active or irregularly active physical activity	Abdominal or pelvic surgery; pregnancy; metabolic disorders; smoking; neurological; respiratory or cardiac disease; PFD or menstrual dysfunctions
**Both**						
Devreese et al. (29)	Cross-sectional	Europe	C: 40 I: 40	C: 50.9 I: 48.4	Patients were referred by the hospital for an individual pelvic floor exercise program	Subjects with a vaginal, urethral, or bladder infection, neurological disorders, LBP or pregnancy
Thompson et al. (38)^b^	Cross-sectional	Australia	C: 13 I: 13	C: 37 I: 38	Inclusion criteria for both groups were women aged 20-55 y, premenopausal or on HRT, and using a consistent PFM exercise technique	History of urinary tract or vaginal infection; known or suspected pregnancy; surgery involving incision of the abdominal wall; obesity; history of LBP or sporting activities; neurological disorders; inability to understand English
Madill et al. (31)	Cross-sectional	Canada	C: 28 I: 44	C: 46.8 I: 49.65	C and SUI women aged 21-60 y; not pregnant and not given birth in the last 12 m; in good general health.	Previous gynecological or continence surgery, POP greater than stage 2; intrinsic sphincter deficiency; history of DM; neurological conditions; autoimmune CT disorders; use of any medications to treat or known to exacerbate UI
Arab et al. (24)	Cross-sectional	Asia	C: 10 I: 10	C: 41.66 I: 38.47	Women who had UI, were premenopausal, or were on HRT. Asymptomatic females, matched in age and body mass index and with no symptoms of UI.	Pregnancy and parturition in the previous 12 m; neurological or respiratory disorders; severe LBP; POP greater than stage 2; surgery of the abdominal or pelvic regions
Tajiri (36)	Cross-sectional	Asia	C: 25I: 7	C: 45.8 I: 50.1	Primiparous women	Not reported
Ptaszkowski et al. (34)	Cross-sectional	Europe	C: 14 I: 16	C: 66.1 I: 63.9	Control group: no history of SUI; UI group: history of SUI.	Inability to contract the PFMs; previous gynecological and abdominal surgery; neurologic condition; contraindications to measurements such as infection, menstruation, and allergy to nickel; other symptoms of PFD

a Thompson et al. study (37).

b Thompson et al. study (38).

Abbreviations: NR: not reported; C: continent; I: incontinent; No. number; PFM: pelvic floor muscle; PFD: pelvic floor dysfunction; PFMT: pelvic floor muscle training; AbM: abdominal muscle; LBP: low back pain; HRT: hormone replacement therapy; DM: Diabetes mellitus; UI: urinary incontinence; POP: pelvic organ prolapse; CT: connective tissue; y: years; m: months; pp: postpartum; SUI: stress urinary incontinence; cm: centimeters; kg: kilograms; AHT: abdominal hypopressive technique; BMI: body mass index; cm: centimeters

**Table 2:** Study characteristics related to population, co-activity, and assessed outcomes.

Author	Instruction of co-activity	Maximal voluntary contraction (PFM)	Measurement of correct PFM contraction	Position tested	Measurement of contraction	PFMC Measurement and variable assessed	Muscles tested
**With Pelvic Floor Dysfunction**							
Bø et al. (27)	Activity of PFMs during TrA contraction.	NR	Inward lifting and squeezing of the pelvic openings and vaginal palpation.	Standing	US	Axial plane of minimal hiatal dimensions. Area measured as cm^2^.	PFMs
Tajiri et al. (22)	Recorded TrA during PFM contraction.	Yes	Verbal orientation	Supine	US	Not applicable	AbMs
**Without Pelvic Floor Dysfunction**							
Bø et al. (26)	Activity of the RA during PFM contraction.	Yes	Perineal and vaginal palpation; observation of movement and vaginal pressure measurements.	NR	EMG	Balloon catheters. Result specifications not described.	AbMs
Bø et al. (25)	Activity of PFMs during abdominal contraction.	NR	Vaginal palpation; observation and vaginal pressure measurements.	Supine	EMG	Needle EMG. Result specifications not described	PFMs
Sapsford et al. (6)	Activity of the TrA, RA, OI, OE and PFM was recorded during PFM contraction in three different lumbar spine positions.	Yes	Vaginal palpation.	Supine	EMG	Intravaginal probe using NEEN HealthCare. %MVC-normalized EMG amplitudes.	AbMs
Neuman et al. (33)	Activity of the TrA, OI, and PFM was recorded. The subjects performed PFM and abdominal contraction.	Yes	Perineal and vaginal palpation; observation of movement and vaginal pressure measurements.	Supine and standing	EMG	Vaginal surface EMG. %MVC-normalized EMG amplitudes.	PFMs and AbMs
Madill et al. (9)	Activity of the TrA, RA, OI, OE and PFM was recorded during PFM contraction.	Yes	Squeezing around the vagina and visible cephalad movement of the perineum, without breath holding.	Supine	EMG	Modified Femiscan™ EMG probe. %MVC-normalized EMG amplitudes.	AbMs
Thompson et al. (37)^a^	Activity of the RA, OI, OE, and PFM was recorded during PFM contraction and valsalva.	Yes	Vaginal palpation.	Supine	EMG	Intravaginal probe Using NEEN HealthCare. %MVC-normalized EMG amplitudes.	PFMs and AbMs
Madill et al. (32)	Activity of the TrA, RA, OI, and OE was recorded during PFM contraction.	Yes	EMG and pressure and observation of the perineum.	Supine, sitting, and standing	EMG	Modified Femiscan™ EMG probe. %MVC-normalized EMG amplitudes.	AbMs
Junginger et al. (30)	Activity of the TrA and PFM was recorded during abdominal and PFM contraction.	No	Confirmed by EMG.	Supine	EMG	Intravaginal probe Periform^®^. %MVC-normalized EMG amplitudes.	PFMs and AbMs
Strupp et al. (35)	Activity of the TrA and PFM was recorded. The subjects performed AHT and PFM contraction.	Yes	Inspection and vaginal palpation.	Supine	EMG	Intravaginal probe Chattanooga Group^®^. MVEA-normalized EMG.	PFMs and AbMs
Chmielewska et al. (28)	Measurement of the TrA and RA during PFM contraction.	Yes	Confirmed by EMG.	Supine, sitting, and standing	EMG	Small diameter intravaginal probe. %MVC-normalized EMG amplitudes.	AbMs
Silva et al. (23)	Activity of the TrA/OI during PFM contraction. Activity of the PFM during TrA/OI contraction.	Yes	Vaginal palpation; orientation on how to effectively contract the PFMs	Standing	EMG	Endovaginal sensor PhysioMed Services^®^%. MVC-normalized EMG amplitudes.	PFMs and AbMs
Ithamar et al. (39)	Activity of the TrA/OI, RA, OE and PFM during AHT	Yes	Verbal orientation on how to effectively contract the PFMs	Supine, standing and quadrupedal	EMG	Intravaginal probe. %MVC-normalized EMG amplitudes.	PFMs
**Both**							
Devreese et al. (29)	PFMs during abdominal contraction.	NR	Inward observation of the perineum and vaginal palpation	Supine	Other	Digital palpation. Scoring system	PFMs
Thompson et al. (38)^b^	Activity of the RA, OI, OE, and PFM was recorded during PFM contraction and the Valsalva maneuver.	NR	Vaginal palpation.	Supine	EMG	Intravaginal probe Using NEEN HealthCare. %MRC-normalized EMG amplitudes.	PFMs and AbMs
Madill et al. (31)	Activity of the RA, OI, and OE was recorded during PFM contraction.	Yes	Visible cephalad movement of the perineum.	Supine	EMG	Modified Femiscan™ EMG probe. RMS-MVC EMG amplitudes.	AbMs
Arab et al. (24)	Activity of the TrA and OI was recorded during PFM contraction.	Yes	Lifting of the bladder base on transabdominal US.	Not reported	US	Not applicable.	AbMs
Tajiri et al. (36)	TrA during PFM contraction.	Yes	NR	Supine	US	Not applicable	AbMs
Ptaszkowski et al. (34)	RA during PFM contraction.	Yes	Confirmed by the physiotherapist.	Standing	EMG	Life-care Vaginal Probe PR-02. RMS-MVC EMG amplitudes.	PFMs and AbMs

a Thompson et al. study (37).

b Thompson et al. study (38).

Abbreviations: NR: not reported; AbM: abdominal muscle; PFM: pelvic floor muscle; TrA: transversus abdominis; RA: rectus abdominis; OI: obliquus internus abdominis; OE: obliquus externus abdominis; EMG: electromyography; US: ultrasonography; IAP: intra-abdominal pressure; MVC: maximal voluntary contraction; MRC: maximal reference contraction; MVEA: maximal voluntary electrical activity; RMS: root mean square.

**Table 3:** Risk of bias assessment of the included studies.

Author	Was the selection of exposed and nonexposed cohorts drawn from the same population?	Can we be confident in the assessment of exposure?	Can we be confident that the outcome of interest was not present at the start of the study?	Did the study match exposure and nonexposure for all variables that are associated with the outcome of interest or did the statistical analysis adjust for these prognostic variables?	Can we be confident in the assessment of the presence or absence of prognostic factors?	Can we be confident in the assessment of the outcome?	Was the follow-up of cohorts adequate?	Were co-interventions similar between groups?
Devreese et al. (29)	Definitely low risk	Probably low risk	Probably low risk	Probably high risk	Definitely low risk	Probably high risk	Definitely high risk	Probably high risk
Thompson et al. (38)^b^	Probably high risk	Probably high risk	Probably low risk	Probably low risk	Definitely low risk	Probably high risk	Definitely high risk	Probably high risk
Madill et al. (31)	Probably low risk	Probably high risk	Probably low risk	Probably low risk	Definitely low risk	Definitely low risk	Definitely high risk	Probably high risk
Arab et al. (24)	Probably low risk	Probably low risk	Probably low risk	Probably low risk	Probably low risk	Probably high risk	Definitely high risk	Probably high risk
Tajiri et al. (36)	Probably high risk	Probably high risk	Probably low risk	Probably high risk	Definitely low risk	Probably high risk	Definitely high risk	Probably high risk
Ptaszkowski et al. (34)	Definitely low risk	Probably low risk	Probably low risk	Probably low risk	Definitely low risk	Probably high risk	Definitely high risk	Probably high risk

b Thompson et al. study (38).

**Table 4:** GRADE evidence profile for cross-sectional studies: women without pelvic floor dysfunction *versus* women with pelvic floor dysfunction*.

No. of participants (studies)	Quality assessment					Summary of findings					Certainty in estimates
						Study event rates MD (SD)		Mean difference(95% CI)	Anticipated absolute effects		OR Quality of evidence
	Risk of bias	Inconsistency	Indirectness	Imprecision	Publication bias	Women without PFD	Women with PFD		Risk in women without PFD*	Risk in women with PFD*	
	**Cocontraction activity of transversus abdominis muscles** when the PFMs contract										
52 (2)	Serious limitation^a^	Nonserious limitation^b^	Serious limitation^d^	Serious limitation^e^	Undetectable	2.5 (0.4)**	2.1 (0.3)**	−0.61 (−1.42 to 0.20)	The mean rate of coactivity of the transversus abdominis muscles was 2.5.	The mean rate of coactivity of the transversus abdominis muscles in the exposed group was on average 0.61 lower (1.42 lower to 0.20 higher).	⊕⊕OO LOW
	**Sensitivity analysis of cocontraction activity of transversus abdominis muscles** when the PFMs contract										
32 (1)	Serious limitation^a^	Serious limitation^c^	Serious limitation^d^	Nonserious limitation^e^	Undetectable	2.5 (0.4)**	2.1 (0.3)**	−1.02(−1.90 to -0.14)	The mean rate of coactivity of the transversus abdominis muscles was 2.5.	The mean rate of coactivity of the transversus abdominis muscles in the exposed group was on average 1.02 lower (1.9 lower to 0.14 lower).	⊕OOO VERY LOW
	**Cocontraction activity of the rectus abdominis muscle** when the PFMs contract										
128 (3)	Serious limitation^a^	Serious limitation^c^	Serious limitation^d^	Serious limitation^e^	Undetectable	6 (4)***	8 (5)***	−2.05 (−6.51 to 2.42)	The mean rate of coactivity of the rectus abdominis muscle was 6.	The mean rate of coactivity of the rectus abdominis in the exposed group was on average 2.05 lower (6.51 lower to 2.42 higher).	⊕OOO VERY LOW
	**Sensitivity analysis of the cocontraction activity of the rectus abdominis muscle** when the PFMs contract										
56 (2)	Serious limitation^a^	Serious limitation^c^	Serious limitation^d^	Nonserious limitation^e^	Undetectable	6 (4)***	8 (5)***	0.89 (-0.03 to 1.82)	The mean rate of coactivity of the rectus abdominis muscle was 6.	The mean rate of coactivity of the rectus abdominis in the exposed group was on average 0.89 higher (0.03 higher to 1.82 higher).	⊕⊕OO LOW
	**Cocontraction activity of the obliquus internus abdominis muscle** when the PFMs contract										
118 (3)	Serious limitation^a^	Serious limitation^c^	Serious limitation^d^	Serious limitation^e^	Undetectable	23 (3)****	18 (2)****	−0.47 (−2.38 to 1.44)	The mean rate of coactivity of the obliquus internus muscle was 0.23	The mean rate of coactivity of the obliquus internus in the exposed group was on average 0.47 lower (2.38 lower to 1.44 higher).	⊕OOO VERY LOW
	**Sensitivity analysis of the cocontraction activity of the obliquus internus abdominis muscle** when the PFM contracts										
26 (1)	Serious limitation^a^	Serious limitation^c^	Serious limitation^d^	Nonserious limitation^e^	Undetectable	26 (18)***	57 (34)***	1.10 (0.27 to 1.94)	The mean rate of coactivity of the obliquus internus muscle was 26.	The mean rate of coactivity of the obliquus internus in the exposed group was on average 1.10 higher (0.27 higher to 1.94 higher).	⊕⊕OO LOW
	**Cocontraction activity of the obliquus externus abdominis muscles** when the PFM contracts										
98 (2)	Serious limitation^a^	Serious limitation^c^	Serious limitation^d^	Serious limitation^e^	Undetectable	30 (5)****	21 (4)****	0.01 (−4.00 to 4.03)	The mean rate of coactivity of the obliquus externus muscle was 30.	The mean rate of coactivity of the obliquus externus in the exposed group was on average 0.01 higher (4.00 lower to 4.03 higher).	⊕OOO VERY LOW
	**Sensitivity analysis of the cocontraction activity of the obliquus externus abdominis muscle** when the PFMs contract										
26 (1)	Serious limitation^a^	Serious limitation^c^	Serious limitation^d^	Nonserious limitation^e^	Undetectable	9 (4)***	37 (18)***	2.08 (1.10 to 3.06)	The mean rate of coactivity of the obliquus externus muscle was 9.	The mean rate of coactivity of the obliquus externus in the exposed group was on average 2.08 higher (1.10 higher to 3.06 higher).	⊕⊕OO LOW

Abbreviations: MD: mean difference; SD: standard deviation; PFD: pelvic floor dysfunction; CI: Confidence interval.

* Cross-sectional studies started from high quality evidence because of the nature of the clinical question.

** The estimated risk control was taken from the mean estimated control risk from the Tajiri (2011) study (35).

*** The estimated risk control was taken from the mean estimated control risk from the Thompson (2006b) study (37).

**** The estimated risk control was taken from the mean estimated control risk from the Madill (2009) study (30).

a Issues related to exposure and outcome assessments, follow-up period and cointerventions.

b There may not be considerable heterogeneity (I^2^ <50%).

c There is considerable heterogeneity (I^2^>75%).

d Included studies with only one PFD.

e 95% Confidence interval for absolute effects include clinically important significance and no significance.
